# Improving Odometric Accuracy for an Autonomous Electric Cart

**DOI:** 10.3390/s18010200

**Published:** 2018-01-12

**Authors:** Jonay Toledo, Jose D. Piñeiro, Rafael Arnay, Daniel Acosta, Leopoldo Acosta

**Affiliations:** Computer Science and System Department, Universidad de La Laguna, 38200 Santa Cruz de Tenerife, Spain; jpineiro@ull.es (J.D.P.); rarnayde@ull.es (R.A.); daniel.leo.ah@gmail.com (D.A.); lacosta@ull.es (L.A.)

**Keywords:** autonomous vehicles, odometry, neural networks, Robotics

## Abstract

In this paper, a study of the odometric system for the autonomous cart Verdino, which is an electric vehicle based on a golf cart, is presented. A mathematical model of the odometric system is derived from cart movement equations, and is used to compute the vehicle position and orientation. The inputs of the system are the odometry encoders, and the model uses the wheels diameter and distance between wheels as parameters. With this model, a least square minimization is made in order to get the nominal best parameters. This model is updated, including a real time wheel diameter measurement improving the accuracy of the results. A neural network model is used in order to learn the odometric model from data. Tests are made using this neural network in several configurations and the results are compared to the mathematical model, showing that the neural network can outperform the first proposed model.

## 1. Introduction

Odometry is one of the basic localization systems in any autonomous vehicle [1,2]. It is based on the use of data from on-board sensors in order to estimate changes in position and orientation from the vehicle itself, and is subsequently used in many autonomous systems to estimate their position relative to a starting location, by integrating sensors measurements. However, this method is sensitive to errors due to the integration over time and the final position is usually not very accurate. Usually, the odometry output of a robot is very poor, it is only valid for a few meters, and needs others sensors to obtain a good localization system. Any small increment in odometry accuracy can improve the whole localization system a lot.

Usually, odometry is used in combination with positioning systems like GPS, lasers, radio frequency markers, natural or artificial beacons, and others [3]. When mechanical odometry is not available, visual odometry can be used [4] to estimate vehicle position from changes between images. A complete sensorial system for an autonomous vehicle is based on multiple sensors combined to get position and orientation [5,6]. Some algorithms used for this purpose are Kalman filters [7], particle filters based on Montecarlo simulation [8], etc. Sensors excluding odometry usually need external information obtained from the environment, so in many situations these sensors simply do not work correctly. For example, GPS loses coverage when the vehicle does not have a full sky vision [9]. As another examples, if we introduce beacons, we need to structure the whole environment, or a laser needs available features to recognize in the environment, and these features should be in the range of action of the laser (typically between 10–20 m) [10].

The main advantage of odometry is that all localization information comes from the robot itself. Odometry information is always available and usually it is the only localization information when other sensors are not able to provide data, so a good odometry based localization system is always necessary and it is usually the first step to localization [11,12], obstacle detection [13], and navigation [14].

In wheeled robots, odometry is based on the movement of each wheel. A rotation sensor (rotation optical encoder) is attached to each drive wheel of the robot, and, knowing the wheel diameter, it is possible to approximate linear displacement of each wheel. Using each wheel traslation and the separation between wheels, position, and orientation of the robot (pose) is calculated. All of the calculation is based on optical encoder information, which obtains, in real time, the rotation angle of each wheel. The sensor and all of the parameters can be affected by errors, so final pose based on odometry usually is very noisy. For more details, see Section 2.

The main disadvantage of odometry is incremental error; odometry starts in a known pose, and this pose is updated with small increments using the integration of information acquired from sensors. Errors grow very fast due to the integration of sensor data, so a continuous calibration system is crucial.

**Figure 1:**
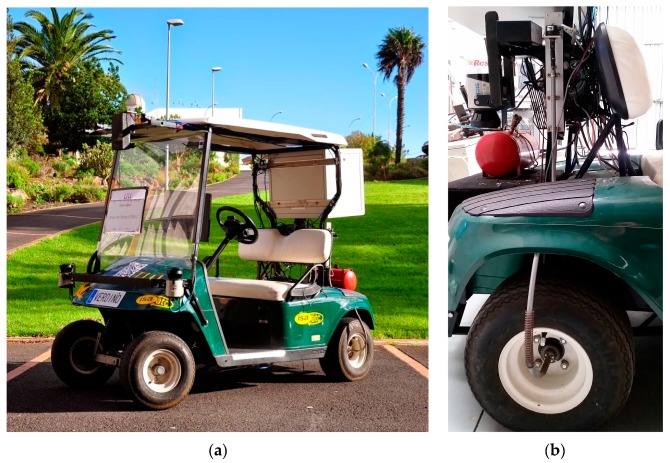
(**a**) Verdino autonomous golf cart; and, (**b**) odometric sensor based on a flexible link from the center of the wheel to the encoder placed in the vehicle.

In this paper, an intelligent odometry system for an autonomous vehicle is presented. The VERDINO project, Figure 1, is being developed at La Laguna University Robotics Group (GRULL) in Tenerife (Spain). The project is aimed to navigate in ITER (Technological Institute for Renewable Energies) facilities in Tenerife.

The project consists of an electric vehicle, a standard EZGO golf cart, electronically and mechanically adapted for computer control, aimed for people transportation within a bioclimatic housing development. In the bioclimatic resort, no combustion cars are allowed, so all of the transport is made based on electric vehicles. The objective of the project is to build an on-demand autonomous transportation system, so the inhabitants of the village can use it as the internal transportation system between the houses and the central parking. The users have a request button in each house, so the car will go in an autonomous way to their entrance. The user rides on the car and selects its destination between available places, so the golf cart can drive to that destination, leave the passenger, and come back to the central station. The autonomous cart works like an internal taxi inside the bioclimatic area.

This vehicle features the following systems:

- Optic encoder based odometry system.
- Centimeter GPS system.
- Multiple LIDAR units.
- Stereo camera.

Based on this hardware infrastructure, the following features and capabilities have been developed:

- Vision algorithms for non-structured road edge detection and obstacle detection.
- Stereoscopic vision algorithms for object detection (in particular, pedestrians and animals).
- LIDAR map based localization.
- Obstacle detection and avoidance module.
- Navigation module with local and global objectives.
- Telemetry system to detect possible failures from the base station.
- User interface to set destination.

The vehicle is able to navigate autonomously in unstructured environments using the sensors that were located in the prototype, but there are situations in which no sensor besides odometry can provide information, so improving the odometry system will improve the full system performance. The localization subsystem of the prototype is based on odometry, the rest of sensors are included to improve accuracy when their information is available.

Other sensors, like gyroscopes, can be used in order to complement odometric information and improve accuracy. As examples, in [15], the information of the odometric system is fused with gyro information according to a set of rules; In [16], the rate angle is measurement using a gyroscope and the displacement using only one encoder; In [17], the authors intend to correct odometry errors when the robot is traversing a bump, trying to reduce non-systematic errors and getting robot angle in the bump based only on gyro information. In [18], an Unscented Kalman filter is used to fuse information from odometry and gyroscope, improving final accuracy.

The sensor used to detect front wheels angle is an encoder coupled to the steering wheel, this encoder gives enough information to drive autonomously the cart, but the accuracy of the angle is low. Some problems like the home position of steering angle and non-linearity between steering wheel and steering angle reduce its applicability to improving odometric accuracy. With this sensor, the model for obtaining a position based on speed and steering angle is worse than a badly calibrated odometry, so the authors have discarded it in order to improve odometry.

The main contribution of this paper is the application of a sensor set, including real time wheel diameter measurement to improve the accuracy of the odometric system for a golf cart, or similar Ackerman steering vehicles. The information generated by the sensors is processed using a neural network to include effects, which a static model does not represent. Previous works are based on differential robots with solid wheels that do not include this sensorial system. The application of neural networks to odometry in previous works is based on the detection of errors, and making parameter corrections in a mathematical model. In this paper, a continuous learning system based on neural networks is presented, able to adapt the model to changes in the system. This paper is organized as follows: in Section 2, equations that describe cart odometry are obtained. In Section 3, the model previously obtained is validated with real data. In Section 4, the odometric system is updated to include a wheel radius sensor, and Section 5 presents a neural network based odometry with some variations designed to improve its accuracy.

## 2. Odometric System

The odometric system is based on encoders coupled to rear wheels, as shown in Figure 1. Each encoder provides 1024 pulses per revolution and each revolution of the wheel generates a revolution of the encoder (1:1 coupling). Wheel rotation is transferred to encoders through a flexible mechanical transmission system that goes from the center of each wheel to the encoder placed on the side of the vehicle (see Figure 1). Encoder output is connected to an ad hoc electronics that samples the encoders signal every 0.5 ms. The electronics is designed to measure and integrate the encoder signals and the output is transmitted to the on-board computer every integration period of 20 ms. The integration is made in the microcontroller installed in the ad hoc electronics, based on Euler integration, collecting encoder increments for the integration time.

Kinematic state of the cart (pose) is described by its position (x, y) with respect to a fixed reference system and its orientation θ (angle between X axis of reference system and the cart longitudinal position).

**Figure 2:**
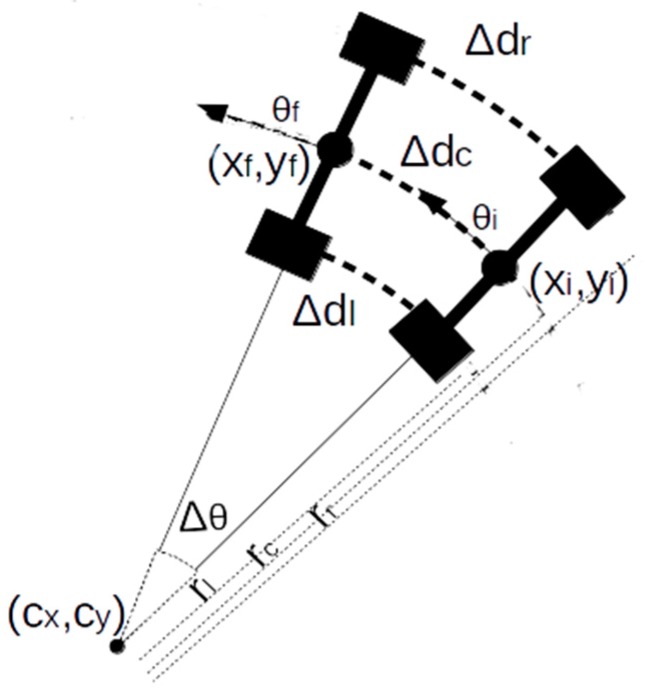
Odometric model.

When the prototype is turning, a circular trajectory is followed. The integration time is small enough to consider the trajectory curvature as constant. In Figure 2, initial (x_i_, y_i_) and final (x_f_, y_f_) position after and integration time are shown. Rear wheels displacements are obtained from the encoders and wheel size.

The arc length of right (*Δ_dr_*) and left (*Δ_dl_*) rear wheels can be calculated based on encoder measurement (*Δ_cr_*, *Δ_cl_*), wheel radius (*R_r_*, *R_l_*), and encoder resolution (*Enc_r_*, *Enc_l_*).

(1) $$\begin{array}{c} \Delta d_{r}=\frac{2\pi R_{r}\Delta c_{r}}{Enc_{r}}\\ \Delta d_{l}=\frac{2\pi R_{l}\Delta c_{l}}{Enc_{l}} \end{array}$$

Curvature radius for each wheel and center is calculated based on wheel distance *d_w_*.

(2) $$\begin{array}{c} r_{r}=\frac{\Delta d_{r}d_{w}}{\Delta d_{r}-\Delta d_{l}}\\ r_{l}=\frac{\Delta l_{r}d_{w}}{\Delta d_{r}-\Delta d_{l}}\\ r_{c}=\frac{d_{w}}{2}\frac{\Delta d_{r}+\Delta d_{l}}{\Delta d_{r}-\Delta d_{l}} \end{array}$$

With these assumptions, angle *Δ_θ_* and position (*Δ_x_*, *Δ_y_*) increments are:

(3) $$\begin{array}{c} \Delta_{\theta }=\frac{\Delta d_{r}-\Delta d_{l}}{d_{w}}\\ \Delta_{x}=r_{c}\left(\cos\left(\theta \right)\sin\left(\Delta_{\theta }\right)-\sin\left(\theta \right)\left(1-\cos\left(\Delta_{\theta }\right)\right)\right)\\ \Delta_{y}=r_{c}\left(\sin\left(\theta \right)\sin\left(\Delta_{\theta }\right)+\cos\left(\theta \right)\left(1-\cos\left(\Delta_{\theta }\right)\right)\right) \end{array}$$

and in the last step, position and orientation are updated with the last computed increments:

(4) $$\begin{array}{c} \theta_{i+1}=\Delta_{\theta }+\theta_{i}\\ x_{i+1}=\Delta_{x}+x_{i}\\ y_{i+1}=\Delta_{y}+y_{i} \end{array}$$

The odometry model only depends on three free parameters, wheel radiuses (*R_r_*, *R_l_*) and wheel separation *d_w_*. According to this model, the only step to tune the odometric system is to measure these parameters as accurately as possible.

## 3. Odometric Validation

In order to validate equations presented in Section 2, some tests are made using our platform Verdino. A differential GPS is used as a ground truth, and data from odometry synchronized with GPS positioning is obtained. The GPS is a centimetric DGPS (a JAVAD GNSS Triumph-14, JAVAD GNSS Inc., Rock Avenue, San Jose, CA 95131, USA) with a horizontal precision below 1 cm and a vertical precision of around 1.5 cm using Differential GPS (DGPS) at a 5 Hz frequency. DGPS gives real time information about position accuracy; this information is calculated based on the number of available satellites, and position optimization results. In order to get the ground truth, the data is captured with good weather conditions with clear sky. The data is analyzed and if some error is detected, then this dataset is discarded. The data set used in this study has an error less than 1.5 cm in the entire traveled path.

The calibration of mobile robots odometry is a well-studied problem. In [19], a study of odometry error sources is presented, classifying errors in systematic due to errors in parameters and non-systematic due to wheel slippage and similar errors. A method to correct systematic odometry errors in mobile robots, based on a known indoor circuit is presented in [20]. The final position, and the ideal final position are compared, and the parameters are adjusted offline to minimize odometry errors. This method works well in controlled situations, with indoor circuits with smooth surfaces and curves, where nonsystematic errors are minimized. However, in outdoors, external environments, where non-systematic errors can appear, the circuit is not even or smooth, it is more convenient a continuous pose ground truth calibration as presented below.

**Figure 3:**
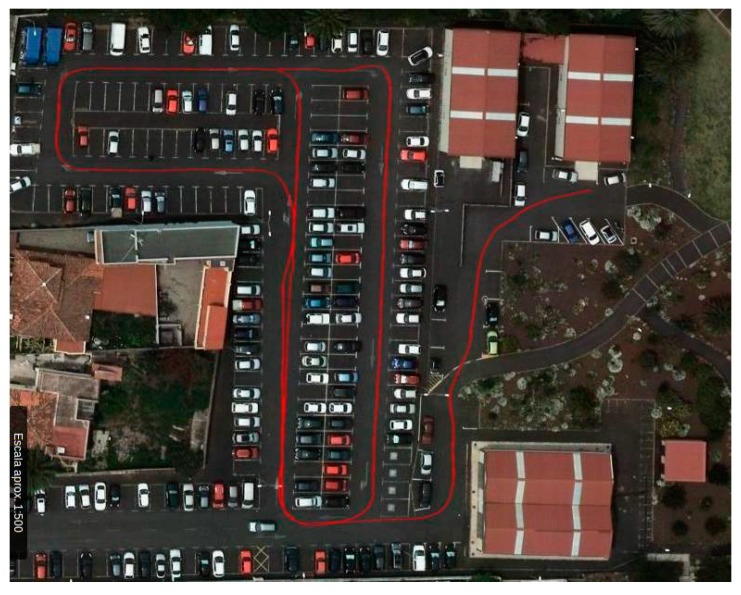
Parking of Computer Science in University of La Laguna, in red the actual path traveled in the experiments.

Figure 3 shows an image of the Computer Science parking at University of La Laguna where tests were made. The red line shows the actual path of the vehicle based on GPS sensor. The parking has a slope of 10 degrees and some curves; these features allow for us to reproduce in the tests many possible situations present in a real road.

The first tests were made using the model presented in Section 2. A least square optimization of wheel radiuses (*R_r_*, *R_l_*) and wheel distance *d_w_* is made in order to get the best static parameters in order to minimize global error. The function to minimize is shown in Equation (5).

(5) $$\begin{array}{c} min\\ R_{l},R_{r},d_{w} \end{array}{\left\|f\left(R_{l},R_{r},d_{w}\right)\right\|}^{2}=\begin{array}{c} min\\ R_{l},R_{r},d_{w} \end{array}{\left(\begin{array}{c} x_{\left(i+N\right)}-xGPS_{\left(i+N\right)}\\ y_{\left(i+N\right)}-yGPS_{\left(i+N\right)} \end{array}\right)}^{2}$$

In order to get data for minimization, the position is started at current position (*x_i_*, *y_i_*), and evolved *N* steps using data from encoders, according to Equation (4). Odometric and GPS data are synchronized, so error is obtained when comparing the position after evolving the odometry pose (*x_i+N_*, *y_i+N_*) with the real position N steps away from the gps data (*x_GPSi+N_*, *y_GPSi+N_*). This error is used to tune the free optimization parameters (*R_r_*, *R_l_*, *d_w_*). This process is made for all of the points in the data set, getting the parameter values which minimize the global error.

The number *N* of iterations is fixed to 250, this distance is 5.5 m in average with a maximum distance of 10.3 m which is a medium distance in order to calculate the error. In 250 iterations, the distance traveled by the cart is not too small so the error between odometric position and GPS position is appreciable and not so big that the influence in each parameter is blurred due to the long path traveled and compensating errors at the final position. In Table 1, the measured and calculated parameters are shown. The results from the optimization process are quite similar and just fix small imprecisions in the odometry building process; however the difference in the final trajectories with these parameters is clear.

**Figure 4:**
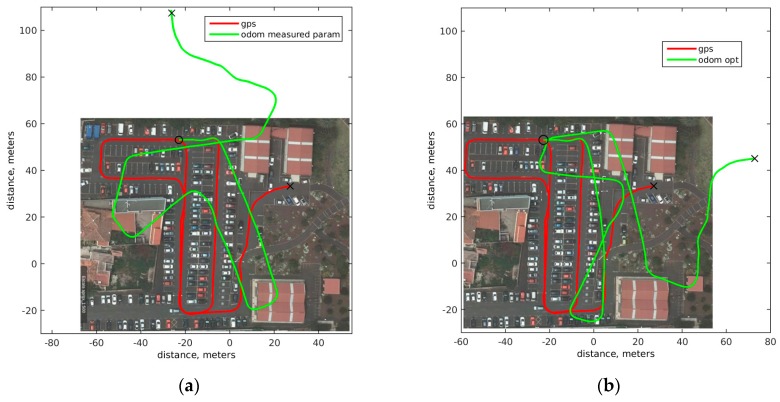
Optimization model results, solid red ground truth based in GPS. (**a**) solid green measurement parameters; (**b**) solid green optimization results.

Results from measurement and estimated parameters are shown in Figure 4, where red plot represents ground truth. The starting point is marked by an O and the final point with a X. The green solid plot in left represents position evolution using the model described in Section 2 and the measured parameters from Table 1. Results are reasonable in straight path sections, but when Verdino takes a curve, the error increases quickly. This result is not a bad result, odometry is only valid for a few meters and outputs like this are expected in this kind of scenarios, due to parameter change dependence an error accumulated in the integration phase. As expected, odometry gives good positions when the displacement is small, but error increases rapidly. Odometry based on optimized parameters is shown Figure 4 right. Behavior is better and the final position of ground truth and odometry is close but still not as good as desired. The error between ground truth and odometric pose based on measurement parameters is 2.3204 × 10^7^ m. This error is calculated as the sum of all the differences between odometric position and ground truth, so it includes the accumulative error in the whole path. In the case of optimized parameters the error is 1.1444 × 10^7^ m using the same method, as expected the error is reduced significantly. In the parameter measurement experiment, the difference between ground truth and odometric pose after 1 s evolution is in average 0.2431 m, and 1.234 m after 5 s. For optimized parameters, this error for 1 s is 0.1976 m and for 5 s is 0.9305 m.

This test shows that the model presented in Section 2 is not good enough to represent all of the effects that are present in cart odometry. Wheels are not rigid solids, so their radiuses change according to road conditions and the forces exerted. Changes in wheel diameters that cannot be described in the model are: changes in wheel diameters due to change in inclination during curves, loss of pressure of wheels, or changes in distribution of weight in the cart. All of these effects cannot be reflected in the model and can reduce the accuracy of results.

## 4. Wheel Radius Model

**Figure 5:**
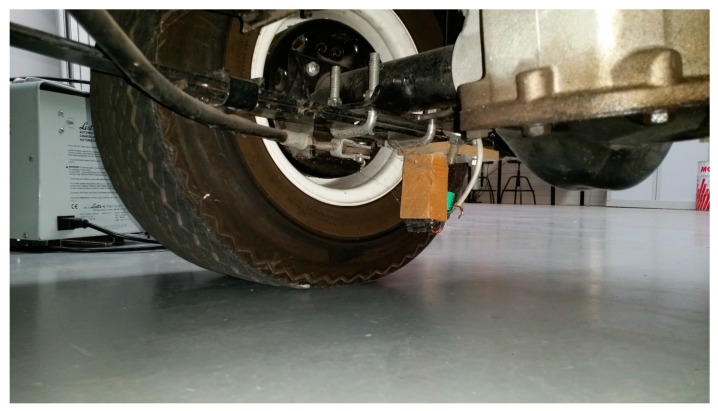
Range sensor used to get a real time radius measurement used to improve odometry accuracy.

**Figure 6:**
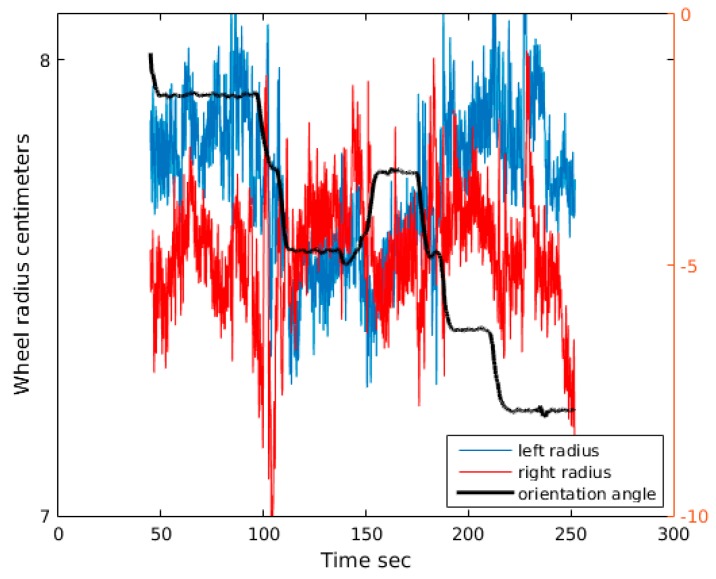
Wheel radius for left (blue) and right (red) wheels. In black is the orientation angle of the cart in radians.

In order to improve accuracy results, a new sensor is included in the system to measure in real time wheel radius Figure 5. The range sensor is based on a Sharp short range optical sensor (2D120X measurement between 4~30 cm) placed close to wheel shaft and pointing to ground at a distance of 8 cm. The range sensor gives a measurement each 40 ms. The sensor is installed under the vehicle, so it is not affected by sun light. It includes an output capacitor to reduce electronic noise. Sensor output is shown in Figure 6, the output oscillation is due to vibrations and road surface rugosity. Using this sensor, a synchronized measurement of the radiuses can be made in real time. The sensor installed in Verdino is shown in Figure 5 and the output data representing wheel distance to the floor in Figure 6. Sensor is connected to an ad hoc electronics that gets the floor distance to sensor and calculates the wheel diameter. The sensor has millimetric resolution and is placed close to the ground in order to improve accuracy.

As seen in Figure 6, the wheel radius is far from being a constant and depends on the current cart maneuver. For example, when the cart is turning left, approximately at 100 s. in the chart, the right radius increases and the left radius decreases. This small change in wheel sizes is not included in the previous model, so integrating this information in the model, a similar optimization process like that described in Section 3 can be carried out, but now including two new parameters to account for the wheel radius changes.

**Figure 7:**
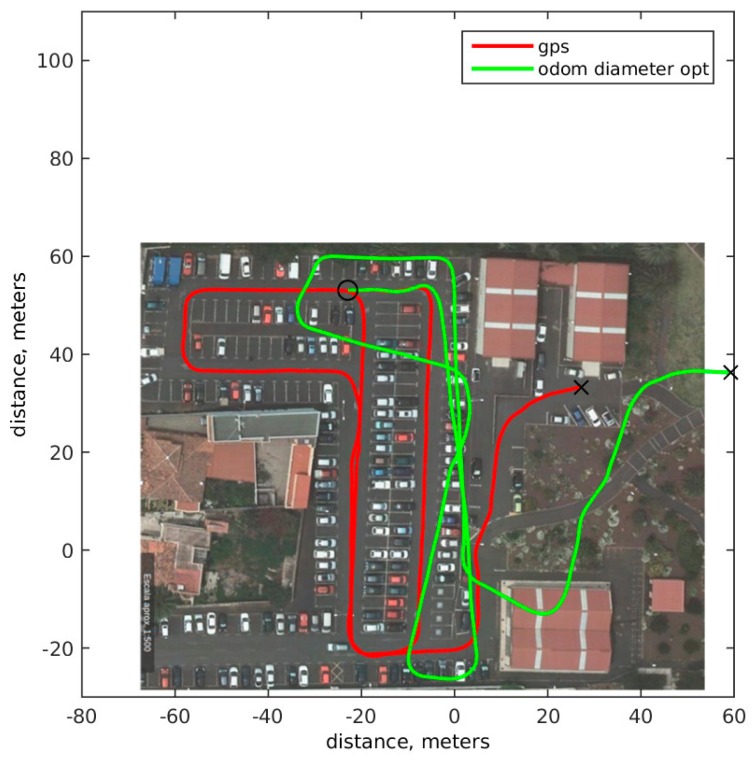
Optimization model including wheel radius change sensor; solid red ground truth based GPS, green, optimization result including radius.

Figure 7 shows in solid green the optimization including the real time wheel radius calculation. Results are better with radius as expected, so information of wheel radiuses increases accuracy in final position calculation. As seen in chart, the main difference between the two techniques is in the turns, straight paths look quite similar, but turns are better represented including wheel radius changes. The error for odometric based optimization accumulated for all points is 1.1444 × 10^7^ m, and when wheel radius changes are considered the error is 4.8561 × 10^6^ m, giving a 56% reduction. The average difference between position in ground truth and the radios optimization after 1 s of evolution is 0.1972 m and after 5 s is 0.9147 m.

It is clear that including a sensor to measure wheel radius improves the odometric localization, but this sensor has not yet enough accuracy to be the only navigation sensor. In order to improve results, new techniques are tested and compared with model based optimizations.

## 5. Neural Network Model

Another way to face of the odometry problem is to avoid calculating a mathematical model from cart dynamic equations, but instead learning a model based on data. This kind of models are very flexible and can take into account other factors which affect odometry and are not considered in a static mathematical model like that presented in Section 2. Some effects that can be reflected by a learning model are speed, turn direction, changes in spin rate, wheel slippage, etc.

Neural Networks are learning algorithms inspired by biological neurons [21,22]. Networks can be used for learning nonlinear complex mathematical functions from data. The function to learn is represented by a set of artificial neurons that receive an input and compute an output as weighted product sums. The weights are computed on a training phase where network output is compared to desired output. Function gradient changes are used to tune weight from various iterations until error reach a minimum or maximum number of iterations are reached. Neural networks are a general interpolator, so with careful training a good generalization can be obtained.

Other authors have used neural network in order to improve odometry accuracy, as in [23] where a neural networks is used to correct the errors generated in simulation by a mathematical fixed odometric model. In [24], neural networks are used to estimate the odometry error of a mobile robot.

**Figure 8:**
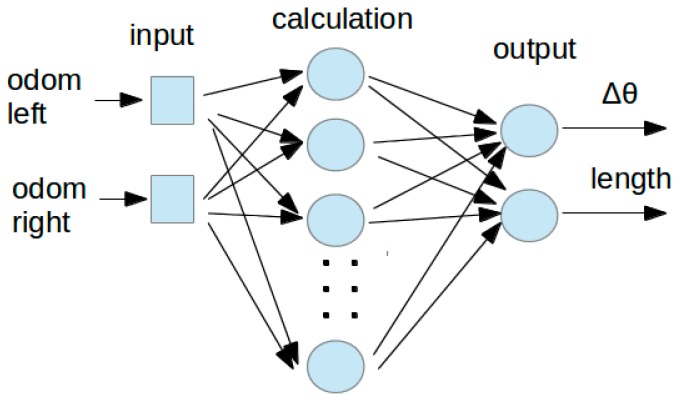
Feed-forward neural network schematic for odometry.

In this paper, a feed-forward neural network is trained using data from odometry and GPS data is used as a ground truth. The network is shown in Figure 8. It has two inputs, the incremental counts between current and previous iterations of left and right encoders from the odometry system. The activation function is a sigmoid; the training set is composed of 7100 encoder data points with their correspondence ground truth based on GPS; the validation set is composed of 3000 points from the same path, but these points are not used to train the network. The tests are made with other data set of 11,500 points in the same scenario. The data of the GPS is interpolated to get the current GPS position of the cart when the odometry sensor sends encoder data, so odometry and GPS data are based on the same clock. The initial training is made off line, so all of the data is available in order to get interpolated positions.

The network outputs angle change (*Δ_θ_*) and length traveled by the cart due to change in odometry sensors. Therefore, the trained neural network works like a modeling function that gets odometry as input and gives displacement and angle as output.

The network is trained using a Levenberg-Marquadt supervised algorithm with a mean squared error. It has two neurons as inputs, fifty hidden neurons and two outputs. The training dataset is obtained from the global dataset, getting the angle increment and displacement due to each odometric input. This data is used to train the network without any other information.

Using neural network output, Equation (6) is applied in order to calculate pose (*X*, *Y*, *θ*). Neural model gives the incremental angle and the displacement. In order to calculate the final pose, all of the incremental outputs of the network are integrated.

(6) $$\begin{array}{c} \theta_{i+1}=\Delta_{\theta }+\theta_{i}\\ X_{i+1}=X_{i}+length\cdot \cos\left(\theta_{i+1}\right)\\ Y_{i+1}=Y_{i}+length\cdot \sin\left(\theta_{i+1}\right) \end{array}$$

**Figure 9:**
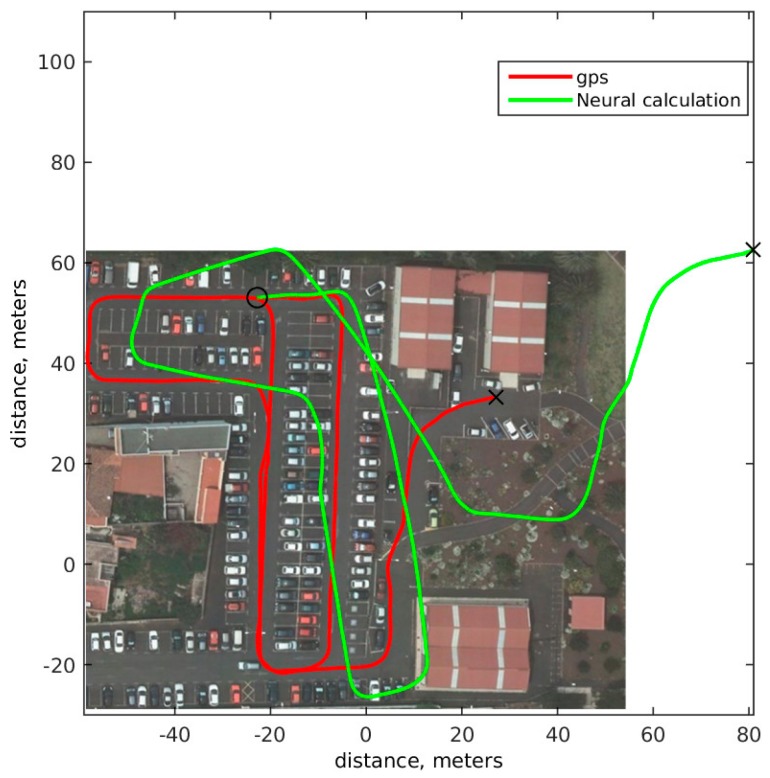
Odometric model based on neural network. Solid red ground truth GPS route. Solid green neural network odometric model path.

Figure 9 shows in solid green results of applying neural model as odometry calculation. Final position is worse than the model based optimization, but during the path, neural computation is closer to ground truth than the model optimization. The accumulative error for neural computation is 1.0361 × 10^7^: slightly smaller than the one obtained with the model based optimization, which is 1.1444 × 10^7^. The average difference between ground truth and neural network position after 1 s is 0.1843 m, and after 5 s is 0.8192 m. This results shows that the learning based model can give better results than many mathematical models, even when only uses input and output snapshot, and not including any historical or state information.

**Figure 10:**
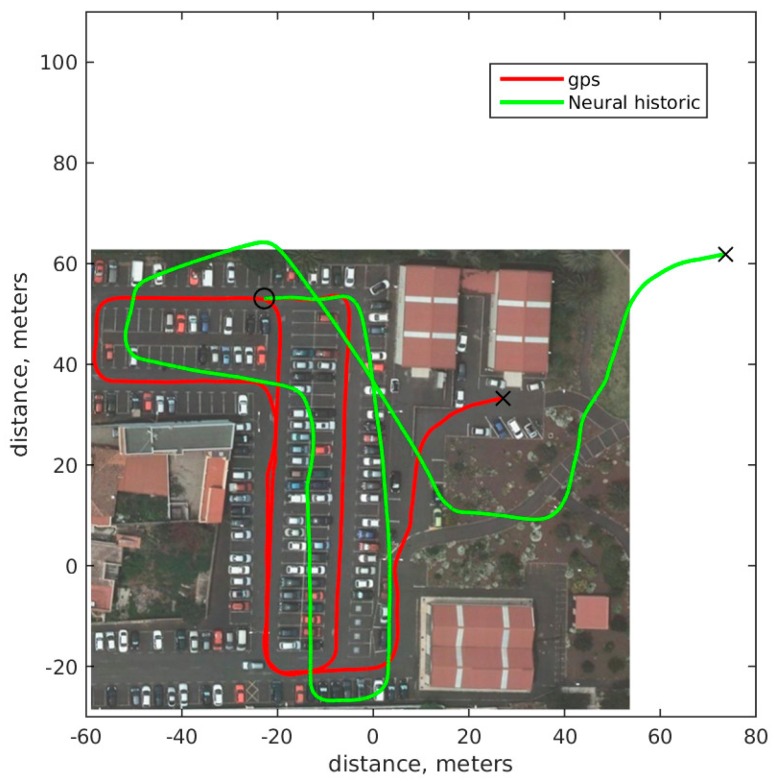
Neural model trains including history information. Solid red ground truth GPS route. Solid green historical neural calculation route.

In order to increase the accuracy in pose calculation the previous neural network is modified by including some historical information about the path traveled by the cart. The new network has four inputs: odometry increments from left and right wheels from current and previous measurements. This information gives the network more information to generate the odometry function, including speed information and acceleration. In Figure 10, a path generated by the neural network with historical information is shown. The route at the final position is quite similar, but during the trace, the position is closer to ground truth. The accumulative error for neural historic is 9.8488 × 10^6^, which gives the best results for all of the experiments presented. The average difference between ground truth and neural model including history after 1 s is 0.1839 m and after 5 s is 0.8179 m.

**Figure 11:**
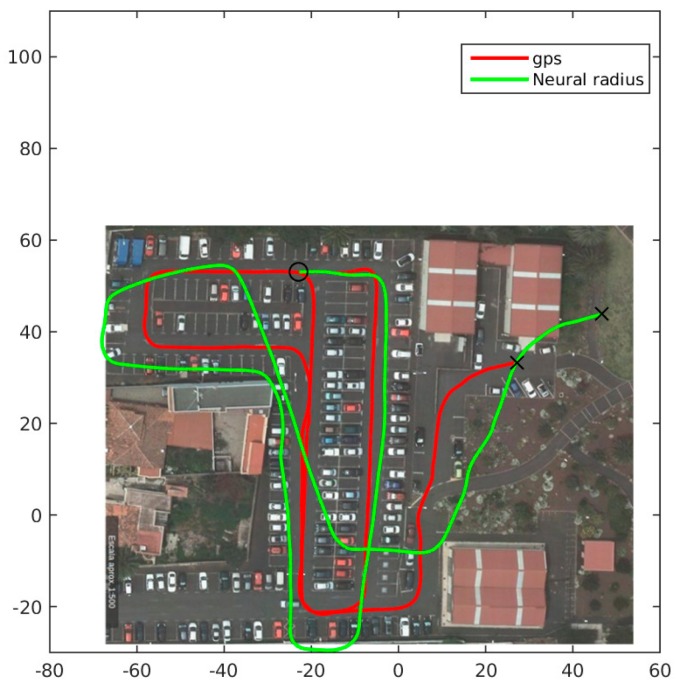
Neural odometric model test, including history information and wheel radius. Solid red ground truth GPS route. Solid green neural calculation route.

A neural network model would increase its accuracy, as more information is available. So, in the last test, a neural network with six inputs is studied. The inputs are encoders from left and right wheels from present and previous measurements and real time wheel diameter obtained from wheel diameter sensor. The network is trained with data output from GPS ground truth. This network has all the information available for optimizing the model. The number of hidden and output neurons of the network is the same than in the previous tests. In Figure 11 the test made with this network is shown in solid green. The path followed based only in odometry information follows pretty good the ground truth. The accumulative error for neural network, including historical information and diameter is 1.4801 × 10^6^, the smallest error from all the tests. The average difference between ground truth and neural odom including diameter and historic information after 1 s is 0.1739 m, and after 5 s is 0.8079 m.

These tests show that neural networks are a very powerful replacement for mathematical models, getting better accuracy and adjusting the behavior of a real system when they have enough information. The neural network can use the relation between inputs and outputs that the mathematical model does not include, getting a very accurate output.

One of the problems of a real odometric system is that model parameters change over time. These changes can be classified as fast or slow changes. Fast changes can be generated by a difference in the weight of the passengers, so suddenly the model changes. Slow changes are due to changes in the characteristics of the environment, like pressure wheel loss, change in temperature, or wheel tear.

A static mathematical model and a learned model cannot face this problem, so the accuracy of the model will decrease along time. To solve this difficulty, a continuous training schema is used in order to get real time actualization of the model. When the cart is navigating, the localization subsystem is used as a source to tune the neural odometric system.

The navigation data (encoders and wheel radius) is included in a historical buffer to re-train the network using the last information in order to maintain an accurate model of the real system. The main problem is to obtain good localization data to train the network. The localization subsystem of the prototype is based on the fusion of multiple sensors and the localization in a map that is built for the prototype [8]. Accuracy in the localization of the cart depends on multiple factors, and localization quality can change depending on sensor information. Monte Carlo Localization (MCL) is a popular technique used to estimate the pose of a mobile robot, using a map to find the actual pose, which allows for the fusion of heterogeneous sensor data. The localization system of the prototype is based on an Adaptive MCL algorithm, which combines data from wheel odometry, an inertial measurement unit, a global positioning system and laser scanning. A particle weighting model that integrates GPS measurements is applied, which increases performance as compared with a particle generation approach. The output of the algorithm is the estimated position and a covariance matrix that measures the localization uncertainty.

The neural network is only retrained to include in the model possible changes in the system when accuracy of the ground truth is bigger (covariance less than 0.5). This schema allows forgetting a tuned model able to face fast and slow model changes, getting an accurate, real time, adaptive model even when the training data is not always accurate. Each new measurement with covariance less than 0.5 are applied to the neural model once, so the computational cost of retraining the network is very small.

**Figure 12:**
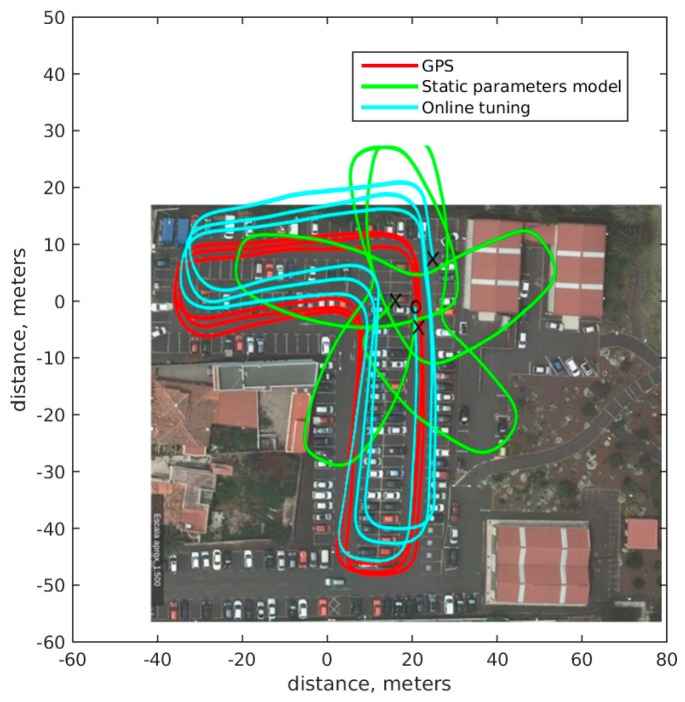
Test after 20 days of tuning odometry. Solid red, GPS based ground truth. green static parameter model. Blue on line tuned neural network model.

In Figure 12, a test made with the prototype 20 days after previous optimization is shown. In solid green, the outputs of the static mathematical model and in solid red GPS based ground truth. Model parameters have changed due to change in temperature, wheel pressure, etc. In Table 2 the measurement parameters, the initial estimated parameters and the current parameters are shown, the change in parameters is small, but due to odometry integration, the pose changes considerably. However, neural network estimation based odometry with on line tuning, in solid blue, is able to adapt to new conditions and get a more accurate result.

## 6. Conclusions

The odometric system of an autonomous vehicle is one of the main sensors for position and orientation estimation in robots and autonomous vehicles. However, its accuracy tends to be small in large distances. In short movements, position estimations are quite precise, but the errors increase quickly and divergences to the ground truth arise when the traveled distance increases. This paper is centered in the odometric system of the autonomous cart Verdino and the solutions applied in order to increase its accuracy.

As first attempt to calculate the position and orientation of Verdino using only odometry, a mathematical model based on the movement equations of the prototype is defined. The main problem of this approach is that many effects that occur on the systems, like wheel diameter or wheel separation changes, are not reflected in the model. To test this model results, a least squares optimization of model parameters are made in order to get the best parameter set. In a second step, and with the objective of improving accuracy, a new sensor is included for measuring wheel radius in real time. This new model increases accuracy, but this is still not enough to be a viable autonomous navigation system based only on odometry.

The parameters set for this model is obtained by an optimization process, so the cart pose based on odometry is close to the best that can be obtained based on this mathematical model and this sensor set. The odometry is well calibrated, so it is necessary another approach to improve the accuracy of the pose. Usually, the odometry output of a robot is very poor, and it needs others sensors to obtain a good localization system. Any small increment in odometry accuracy can improve the whole localization system a lot.

To improve odometry quality, a neural network model is used to represent cart motion. This model has the advantage that learns the input-output relations from data, and can include some effects that a simple mathematical model does not. This neural network model is tested in three variants. In the first a simple input output relation is learned, where the neural network only takes one odometry data point and gives angle and displacement variations. The position accuracy is improved when some historical information is presented, so the model can consider variables like speed or acceleration. To get this historical information, the input of the model consists on the last and the previous odometric information. The best results are obtained when the neural network not only gets odometry information, but real time wheel radiuses, giving the most accurate results. Neural network is continuously trained based on localization information as ground truth when this localization is good keeping updated the model over time.

In Table 3, a results summary is presented. This table is sorted based on the accuracy of the method, and, as expected, the worst behavior occurs when the parameters are measured directly from the cart. In the case where radius information is not available, better results are obtained with neural networks than with mathematical models, and when the network has historical state inputs, the results are even better. When diameter information is available, results clearly improve for both approaches, model, and neural networks, but the neural network outperform the simple model results.

In conclusion, neural network based optimization can be a very powerful system to improve the accuracy of the odometric system, due to its considerations of other effects not included in a mathematical model and its capacity to keep the model update over time. Even small improvements in localization for an odometry system, can improve the whole localization system, and in adverse circumstances, it may be the only available sensor.

## Figures and Tables

**Table 1:** Parameter estimation.

Parameters	*R_r_*	*R_l_*	*d_w_*
Estimated	0.2332 m	0.2295 m	0.978 m
Measured	0.215 m	0.215 m	0.97 m

**Table 2:** Parameter estimation after 15 days.

Parameters	*R_r_*	*R_l_*	*d_w_*
Measured	0.215 m	0.215 m	0.97 m
Initial estimation	0.2332 m	0.2295 m	0.978 m
Current estimation	0.2105 m	0.2095m	0.971 m

**Table 3:** Tests results.

Test	Accumulative Error	Position Error after 1 s	Position Error after 5 s
Measured parameters model	2.3204 × 10^7^ m	0.2431 m	1.234 m
Optimized parameters model	1.1444 × 10^7^ m	0.1976 m	0.9305 m
Neural Model	1.0361 × 10^7^ m	0.1843 m	0.8192 m
Historical Neural Model	9.8488 × 10^6^ m	0.1839 m	0.8179 m
Optimized real time wheel diameter	4.8561 × 10^6^ m	0.1972 m	0.9147 m
Historical wheel radius Neural Model	1.4801 × 10^6^ m	0.1739 m	0.8079 m
